# Ultrasound-Guided Diagnosis and Treatment of Postsurgical Peripheral Nerve Entrapment in Persistent Pelvic Pain

**DOI:** 10.7759/cureus.109210

**Published:** 2026-05-19

**Authors:** Vinicius Adami Vayego Fornazari, Renato Abu Hana, Oswaldo A Guevara Tirado, Ruben G Ortiz Cordero, Grit A Adler

**Affiliations:** 1 Radiology, University of Florida College of Medicine – Jacksonville, Jacksonville, USA

**Keywords:** chronic pelvic pain, genitofemoral nerve, neuropathic pain, peripheral nerve injury, pulsed radiofrequency, ultrasound-guided intervention

## Abstract

Postsurgical peripheral neuropathic pain may be overlooked in patients with chronic pelvic and inguinal pain when overlapping etiologies coexist. This case report describes an ultrasound-guided diagnostic and therapeutic approach for suspected postsurgical neuropathic pain after cosmetic abdominal surgery with persistent pain refractory to conventional pelvic surgical treatments (pelvic veins embolization and endometriosis surgery). A patient with chronic pelvic and inguinal pain underwent focused neuropathic clinical assessment and ultrasound evaluation. Targeted ultrasound identified focal thickening of a superficial nerve in the inguinal region consistent with the genitofemoral nerve. Two reproducible low-volume diagnostic blocks produced complete but temporary pain relief. A stepwise perineural treatment strategy, including hydrodissection and pulsed radiofrequency neuromodulation, was subsequently performed, resulting in sustained pain reduction, functional improvement, and reduced analgesic requirements at three- and six-month follow-ups. This case highlights the potential value of focused neuropathic assessment, ultrasound localization, and diagnostic nerve blocks in selected patients with persistent pelvic and inguinal pain after abdominal surgery. In this setting, ultrasound may also aid in establishing a rapid and precise diagnosis while facilitating minimally invasive, targeted image-guided therapies.

## Introduction

Neuropathic pain is typically characterized by burning or electric shock-like sensations, allodynia, and hyperalgesia [1,2]. Persistent postsurgical pain is common [2,3]; however, the relative contribution of neuropathic mechanisms remains incompletely defined. The genitofemoral nerve provides sensory innervation to the lateral lower abdomen, upper anterior thigh, and genital region and may be vulnerable to injury because of its course near the inguinal region [3,4]. Ultrasound enables visualization of superficial nerves and precise diagnostic blocks, with reproducible pain relief supporting a peripheral neuropathic pain generator [3-5].

This report describes a suspected case of postsurgical genitofemoral neuropathy, an uncommon and potentially underrecognized cause of persistent pelvic and groin pain following lower abdominal cosmetic surgery. We outline a stepwise image-guided diagnostic and therapeutic approach and highlight the potential role of considering peripheral nerve injury in selected patients with persistent pelvic pain.

## Case presentation

A 34-year-old woman developed chronic pelvic and inguinal pain after liposculpture and abdominoplasty that persisted for approximately 10 months despite conservative management. Prior workup included CT and MRI, and venous Doppler ultrasound studies demonstrated pelvic varices and endometriosis. At an outside institution, the patient underwent pelvic variceal embolization without symptomatic improvement. Approximately seven months later, she underwent surgery for endometriosis, including hysterectomy, which also failed to relieve her symptoms.

**Figure 1:**
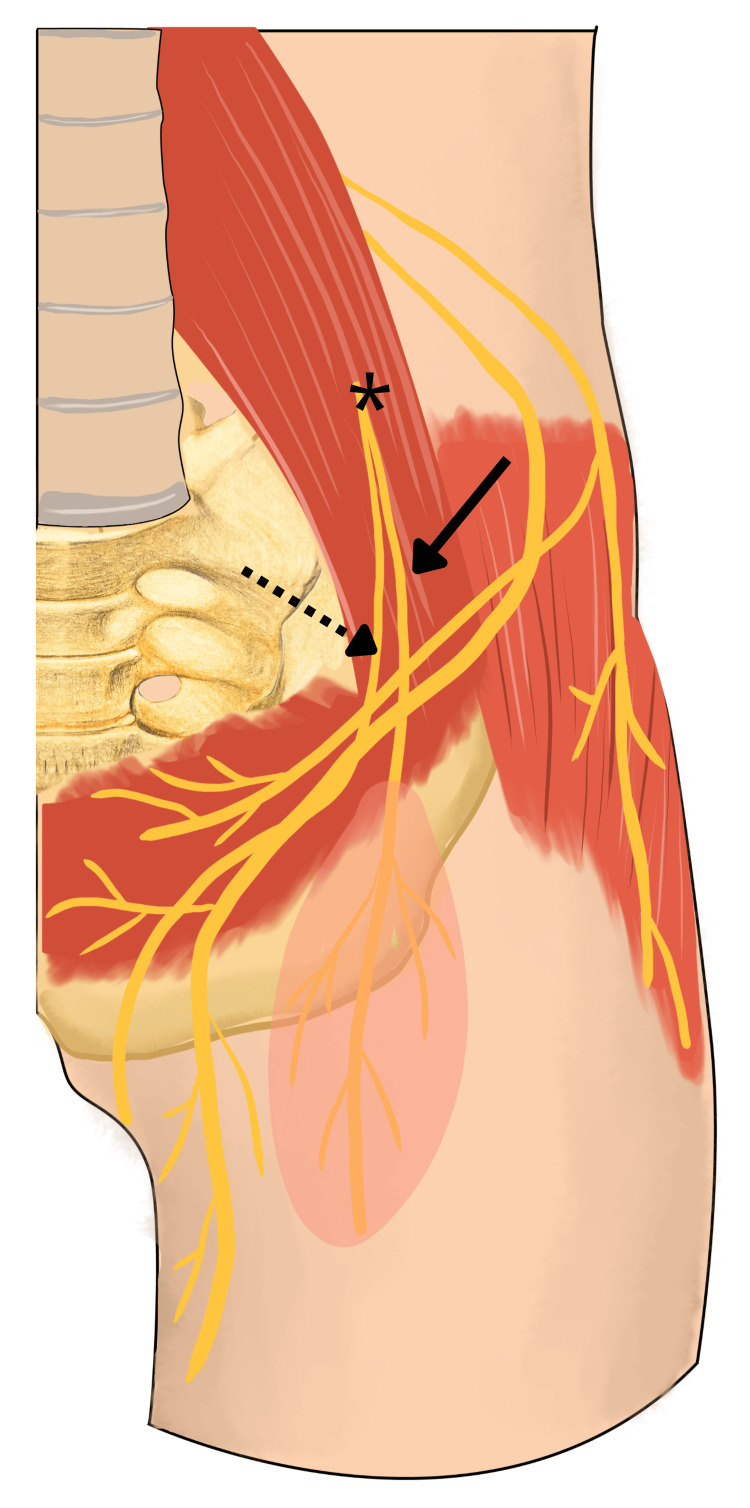
Schematic illustration of the genitofemoral nerve (*) and its femoral (solid arrow) and genital (dashed arrow) branches, correlated with the patient’s pain distribution. The dermatomal pain map (red area) corresponds to the sensory territories of both branches and is associated with a neuropathic pain phenotype and a scar-related positive Tinel sign. The figure was digitally created using Clip Studio (Celsys, Inc., Tokyo, Japan) and enhanced by the authors based on anatomical references of Netter FH, "Atlas of Human Anatomy" (8th ed.) [8].

The patient was reassessed three months later in our institution, and her symptoms were characterized by burning and electric shock-like pain associated with tactile allodynia and hyperalgesia. The patient rated her baseline pain as 7/10 on the numeric rating scale (NRS), with worsening during ambulation [6]. Physical examination demonstrated a focal positive Tinel sign over the right-sided inguinal surgical scar within the topography of the genitofemoral nerve, inferior to the inguinal ligament. The femoral branch was expected to be lateral to the femoral artery in the femoral triangle, whereas the genital branch followed the inguinal canal toward the labial region (Figure 1). Based on a DN4 score consistent with neuropathic pain, the patient’s symptoms were classified as neuropathic rather than somatic [7].

**Figure 2:**
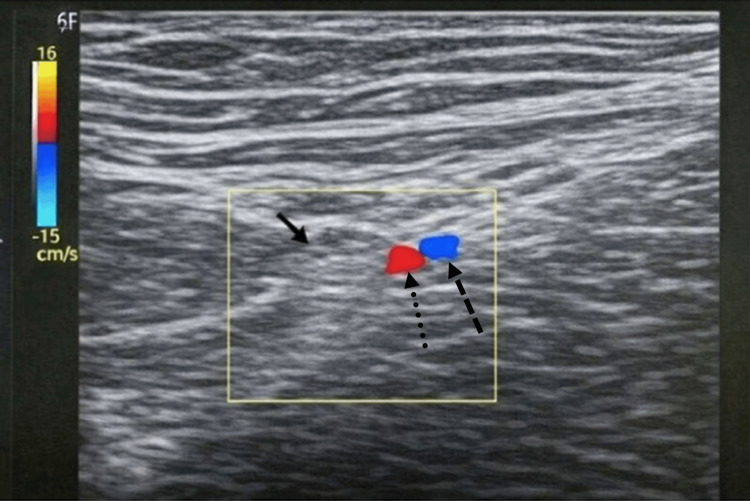
High-resolution short-axis ultrasound image of the right inguinal region demonstrating focal thickening of the genitofemoral nerve (arrow) adjacent to the inguinal ligament and inferior epigastric artery (dotted arrow) and vein (dashed arrow).

Ultrasound (US) was performed with the patient in the supine position using a 12-18 MHz linear transducer and demonstrated focal thickening of the genitofemoral nerve (Figure 2). A targeted ultrasound-guided diagnostic nerve block with 2 mL of 2% lidocaine produced immediate complete pain relief lasting several hours (NRS 1/10). No immediate adverse effects were reported at this time. A confirmatory block performed two weeks later with 2 mL of 2% lidocaine again yielded reproducible complete relief (NRS 0/10), strongly supporting a postsurgical peripheral neuropathic pain generator.

**Figure 3:**
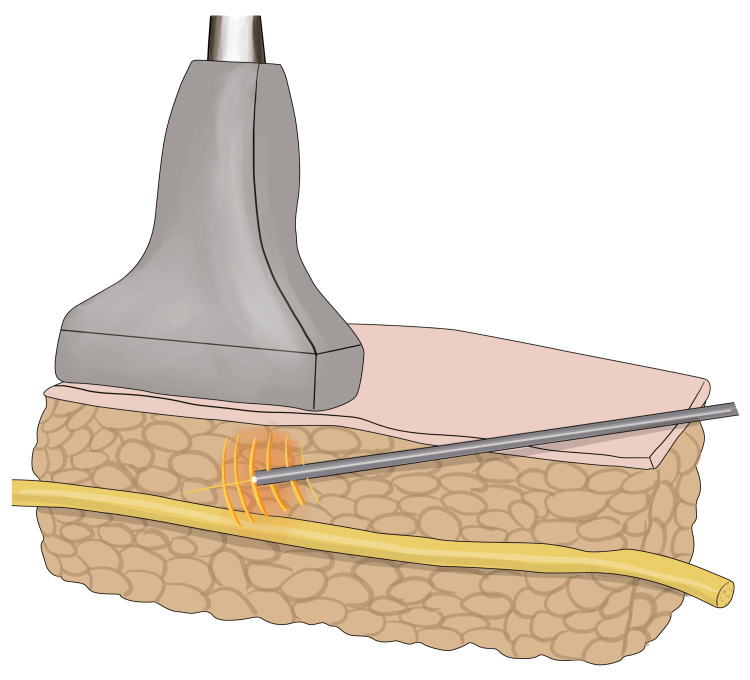
Illustration of an ultrasound-guided pulsed radiofrequency neuromodulation of the genitofemoral nerve, with the needle positioned parallel to the nerve to optimize neuromodulatory energy delivery. The figure was digitally created using Clip Studio (Celsys, Inc., Tokyo, Japan) and anatomically adapted by the authors based on classical neuromodulation theory, with the radiofrequency waves as parallel as possible to the nerve.

A stepwise, US-guided perineural treatment strategy was pursued. Perineural hydrodissection was performed using 2 mL of 2% lidocaine with epinephrine, 2 mL of 5% dextrose, and 4 mL (4 mg) of dexamethasone, resulting in immediate clinically meaningful improvement (NRS 2/10). Because symptoms remained functionally limiting, US-guided pulsed radiofrequency (PRF) neuromodulation was subsequently performed using a 22-gauge Stryker (Stryker Corporation, Kalamazoo, MI, USA) RF cannula with a 5-mm active tip, positioned under ultrasound guidance as parallel as possible to the femoral branch of the genitofemoral nerve (Figure 3). Sensory stimulation confirmed concordant paresthesia before treatment. PRF was delivered in two cycles (20-ms pulse width, 2 Hz, 45 V, and 90 seconds per cycle) while maintaining the temperature below 42 °C.

At three months, the NRS score was 4/10, with partial reduction in analgesic use and functional limitation. At six months, the NRS score decreased to 1/10, with further functional improvement and decreased reliance on analgesics (Table 1).

**Table 1:** Timeline, pain scores, and interventions Timeline of interventions, pain intensity measured using the NRS, and clinical outcomes in a patient with postsurgical neuropathic pelvic/inguinal pain managed with a stepwise ultrasound-guided perineural approach. NRS: Numeric Rating Scale; US: ultrasound

Timepoint (approximate)	Intervention/event	NRS (0-10)	Clinical response
Months before presentation; 10-month pain history	Venous embolization at an outside institution	—	Persistent symptoms
Months before presentation; approximately 7 months after venous embolization	Endometriosis surgery and hysterectomy	—	No improvement in neuropathic pain
Initial presentation; 3 months after last surgeries	Baseline assessment	7/10	Burning/electric pain with allodynia; positive Tinel sign
First diagnostic nerve block	US-guided diagnostic nerve block #1 (2 mL 2% lidocaine)	1/10 (immediate)	Immediate marked improvement in pain intensity
Second diagnostic nerve block (2 weeks later)	US-guided diagnostic nerve block #2 (confirmatory)	0/10 (immediate)	Complete temporary pain relief
Treatment procedure	Perineural hydrodissection (lidocaine + dextrose + dexamethasone)	2/10 (immediate)	Immediate clinically meaningful improvement in pain intensity
Treatment procedure	Pulsed radiofrequency neuromodulation	Decreased vs baseline	Stepwise neuromodulatory treatment
Three-month follow-up	Clinical follow-up	4/10	Partial sustained pain relief, improved function, and reduced analgesic use
Six-month follow-up	Clinical follow-up	1/10	Further sustained pain relief, improved function, and reduced analgesic use

## Discussion

The clinical course of this patient illustrates a diagnostic pitfall in persistent postsurgical pelvic pain. After liposculpture and abdominoplasty, the patient developed persistent burning and electric shock-like pain in the inguinal region, along with tactile allodynia, hyperalgesia, and a positive Tinel sign along the surgical scar. Despite extensive evaluation and treatment for other potential pelvic pain contributors, her symptoms persisted. This sequence highlights how peripheral neuropathic pain generators may remain unrecognized when multiple potential etiologies coexist [1-5].

Persistent postsurgical pain is a well-described clinical entity, affecting approximately 10-50% of patients after major abdominal or pelvic surgery, particularly procedures involving extensive tissue dissection or potential nerve manipulation [2,3,9]. However, these estimates are largely derived from non-cosmetic surgical populations and cannot be directly extrapolated to cosmetic abdominal procedures. Although the true incidence after cosmetic abdominal surgery remains unclear, nerve traction, transection, scarring, and postoperative entrapment are recognized mechanisms across postsurgical cutaneous nerve entrapment syndromes [9]. In this case, focused neuropathic assessment raised suspicion for genitofemoral nerve injury and redirected the diagnostic and therapeutic strategy.

The main diagnostic strength of this case was the concordance among clinical findings, ultrasound localization, and functional confirmation. The US image demonstrated focal thickening of a superficial nerve in the inguinal region consistent with the genitofemoral nerve. In addition, two low-volume ultrasound-guided diagnostic blocks each produced immediate, complete, but temporary pain relief, strongly supporting a peripheral neuropathic pain generator.

Although the ilioinguinal, iliohypogastric, and genitofemoral nerves may all contribute to groin pain and have overlapping sensory territories, several clinical and procedural findings supported predominant genitofemoral nerve involvement in this case [1,9]. Clinically, the patient’s pain distribution was localized to the anterior groin and proximal medial thigh, consistent with the femoral branch of the genitofemoral nerve, rather than the more superior iliohypogastric or inferomedial ilioinguinal sensory territories. US evaluation did not reveal focal abnormalities along the expected course of the ilioinguinal or iliohypogastric nerves, whereas the genitofemoral nerve was identified in a location concordant with the patient’s maximal tenderness. Furthermore, a targeted US-guided diagnostic block at the genitofemoral nerve resulted in immediate and substantial pain relief, supporting its role as the primary pain generator. Some sensory overlap cannot be excluded; however, the concordance of anatomical, clinical, and interventional findings supports a diagnosis of suspected postsurgical genitofemoral neuropathy [1,9].

This diagnostic confirmation allowed progression to a stepwise US-guided treatment approach. Because peripheral nerves are small, ultrasound plays an important role in both the assessment of peripheral nerve disorders and the guidance of targeted perineural interventions [3,4]. Perineural hydrodissection is intended to separate the nerve from surrounding fibrotic tissue or adhesions, potentially restoring nerve mobility and reducing mechanical irritation [4,10].The injectate combined local anesthetic for immediate symptom modulation, dextrose as a commonly used hydrodissection medium, and corticosteroid to reduce perineural inflammation. The total volume was carefully titrated to achieve perineural distribution while limiting excessive spread, particularly given the close anatomical relationship with adjacent groin nerves.

In this case, perineural hydrodissection resulted in immediate clinically meaningful improvement (NRS 2/10), supporting continued treatment and suggesting that ongoing mechanical irritation may have contributed to persistent symptoms. Subsequent PRF neuromodulation was associated with sustained pain reduction at three- and six-month follow-up, with functional improvement and decreased analgesic use. PRF has been proposed as a neuromodulatory treatment that may modulate pain signalling without overt thermal neurodestruction, although its mechanisms and clinical efficacy remain incompletely understood [11].

Collectively, these findings underscore the importance of considering a peripheral nerve pain generator in patients with persistent pelvic or groin pain after abdominal surgery, particularly when treatment of other pelvic etiologies has not resolved symptoms. This case also highlights how focused neuropathic clinical assessment, ultrasound imaging, and functional confirmation with diagnostic nerve blocks may help identify patients who could benefit from targeted intervention.

The main limitation of this report is that it represents a single-case experience without surgical confirmation of nerve injury. Additional limitations include the potential for local anesthetic spread to adjacent nerves, which may confound diagnostic specificity; the possibility of placebo response; the known overlap in sensory territories among the ilioinguinal, iliohypogastric, and genitofemoral nerves; the relatively short follow-up period; and the absence of objective functional outcome measures beyond patient-reported improvement.

Nevertheless, this approach may help identify otherwise overlooked peripheral nerve injuries and guide minimally invasive, image-guided therapies capable of providing durable symptom relief in selected patients. Further studies with longer follow-up are needed to better characterize the durability of pain relief after perineural hydrodissection and PRF neuromodulation in this population.

## Conclusions

Peripheral abdominal nerve injury should be considered in patients with persistent postsurgical pelvic or groin pain with neuropathic features. Ultrasound-guided evaluation enables precise anatomical localization and supports both diagnostic confirmation and targeted interventional management.
